# The Association Between Psychological Distress, Emergency Room Visits, and All‐Cause Mortality Among Colorectal Cancer Survivors

**DOI:** 10.1002/cam4.71107

**Published:** 2025-07-29

**Authors:** Pranali G. Patel, Chaitali Dagli, Nada Al‐Antary, Mrudula Nair, Oluwole A. Babatunde, Nosayaba Osazuwa‐Peters, Poolakkad S. Satheeshkumar, Eric Adjei Boakye

**Affiliations:** ^1^ Department of Epidemiology University of Alabama at Birmingham Birmingham Alabama USA; ^2^ Department of Public Health Sciences Henry Ford Health Detroit Michigan USA; ^3^ Prisma Health Greer South Carolina USA; ^4^ Department of Head and Neck Surgery & Communication Sciences Duke University School of Medicine Durham North Carolina USA; ^5^ Duke Cancer Institute Duke University Durham North Carolina USA; ^6^ Department of Population Health Sciences Duke University School of Medicine Durham North Carolina USA; ^7^ Department of Medicine, Division of Hematology and Oncology University at Buffalo Buffalo New York USA; ^8^ Department of Epidemiology and Biostatistics Michigan State University College of Human Medicine East Lansing Michigan USA; ^9^ Department of Otolaryngology—Head and Neck Surgery Henry Ford Health Detroit Michigan USA; ^10^ Henry Ford Health+Michigan State University Health Sciences Detroit Michigan USA

**Keywords:** colorectal cancer, ED visit, emergency room use, emotional or psychological distress, mortality, unplanned healthcare utilization

## Abstract

**Objective:**

We examined the prevalence of psychological distress and its association with emergency room (ER) usage and all‐cause mortality among colorectal cancer (CRC) survivors.

**Methods:**

We utilized data from the 2000–2018 National Health Interview Survey (NHIS) and the NHIS linked mortality files. The main exposure was psychological distress, assessed with the six‐item Kessler Psychological Distress Scale (K6) and classified as (no/low, moderate, severe). The outcomes were ER usage during the past 12 months and all‐cause mortality. Multivariable logistic and Cox proportional hazards models were used to examine the associations between psychological distress and ER usage and all‐cause mortality, respectively.

**Results:**

A total of 3198 CRC survivors were included in the study, of whom 4.1% and 19.6% reported severe and moderate psychological distress, respectively. Approximately 30% of CRC survivors had ER use, and 41.5% of deaths occurred with a median follow‐up of 84 months. In the adjusted model, compared to CRC survivors with low/no psychological distress, those with severe (aOR = 1.83; 95% CI, 1.10–3.04) or moderate (aOR = 1.60; 95% CI, 1.21–2.10) psychological distress had higher odds of reporting ER use. However, there was no statistically significant association between psychological distress and all‐cause mortality.

**Conclusion:**

CRC survivors with severe or moderate psychological distress have higher ER usage. This finding emphasizes the significance of timely identifying and addressing psychological distress to improve the quality of life and clinical outcomes of patients diagnosed with CRC. Integrating mental health support into routine cancer care may reduce distress levels, potentially leading to fewer ER usages among CRC survivors.

## 1 Introduction

Colorectal cancer (CRC), one of the leading causes of cancer‐related deaths in the US [1], can be managed through a range of medical and surgical options. Treatments for CRC can result in aggressive effects that could leave the patient with long‐term impairment of quality of life (QoL) and psychological distress [2, 3]. CRC survivors face distinct stressors related to the care and complications of ostomy use, gastrointestinal side effects (bowel changes, diarrhea, constipation) following surgical resection, and the multisystem adverse effects of various medical treatment regimens [4, 5, 6]. These persistent challenges could increase the likelihood of social withdrawal, body image disruption, trouble resuming family and work roles, intimacy problems, and marital strain [7, 8], all of which might affect the mental health of CRC survivors [9]. The complexities of cancer‐associated psychological distress extend beyond the initial diagnosis to include the multidimensional aspects of treatment and post‐treatment phases [9]. High levels of psychological distress can negatively impact survivors' health status, QoL, and the quality of care they receive [10]. Furthermore, the burden of psychological stressors can affect individuals' adherence to medication and routine follow‐up appointments, ultimately impacting the frequency of healthcare services usage and overall mortality [11, 12]. As of 2022, there were around 18 million cancer survivors in the United States, 1.4 million of whom were CRC survivors, and this number is expected to increase [13]. The increasing number of CRC survivors emphasizes the importance of addressing psychological outcomes to ensure enhanced QoL among survivors.

Previous studies demonstrated that implementing multi‐level strategies such as individual and group psychotherapy, relaxation training, art therapy, energy conservation training, and psychoeducation to identify and treat psychological distress can significantly benefit patients and improve cancer outcomes [14, 15]. Consequently, further research and clinical efforts are needed to screen for and effectively manage psychological distress in CRC survivors. Despite the consequences of psychological distress, little is documented regarding its prevalence and association with clinical outcomes. This study examined the prevalence of psychological distress and its association with emergency room usage (ER) and all‐cause mortality among CRC survivors. Our findings may help guide future screening and intervention initiatives to improve the lives of CRC survivors.

## 2 Methods

### 2.1 Data Source and Population

We used data from the National Health Interview Survey (NHIS) and NHIS‐linked mortality data, conducted by the National Center for Health Statistics (NCHS) at the Centers for Disease Control and Prevention, including the years 2000–2018. The NHIS was approved by the NCHS Ethics Review Board. Respondents provided verbal consent. The NHIS is a cross‐sectional household interview survey targeting the civilian non‐institutionalized population residing in the United States at the time of the interview. The sample is a probability design, allowing for representative sampling of households and non‐institutional group quarters. We used the sample adult and person questionnaire, which involves random selection, with an increased probability of selection for Black, Hispanic, or Asian individuals aged 65 years or older. The primary aim of the NHIS is to oversee the well‐being of the US population by gathering and examining information on a wide array of health‐related subjects. We also utilized the NHIS‐linked mortality files, which provided 1–20 years of follow‐up, to assess the survival status of NHIS respondents through December 31, 2019. The recommended methodology for linkage was followed [16, 17]. We included adults aged 18–85 years who reported a history of colorectal cancer and had known survival status as of December 31, 2019, from the NHIS‐linked mortality files. Cancer survivors were respondents having any history of CRC based on the American Cancer Society's definition of survivors [18].

### 2.2 Dependent Variables

Our outcomes of interest were ER use and all‐cause mortality. ER use was assessed with the question, “During the past 12 months, how many times have you gone to a hospital ER about your own health?” Based on their responses, participants were categorized as either having no ER use or having any ER use (at least one). All‐cause mortality was recorded as yes or no in the NHIS‐linked mortality files.

### 2.3 Independent Variable

The exposure variable was psychological distress, which was measured using the validated Kessler (K6) scale [19]. Respondents were asked how often in the past 30 days felt (1) so sad nothing cheers them up, (2) nervous, (3) restless/fidgety, (4) hopeless, (5) everything was an effort, and (6) worthless. The scores were on a 5‐point Likert scale which were recoded as “0—None of the time,” “1—A little of the time,” “2—Some of the time,” “3—Most of the time,” “4—All of the time.” Responses were scored and added to produce a range a K6 score of 0–24. We classified the scores into three categories: no/mild (0–4 score), moderate (5–12 score), and severe (≥ 13 score) psychological distress based on previous literature [19, 20, 21].

### 2.4 Covariates

Covariates included in the study were: age at survey, age since CRC diagnosis, gender (male, female), race/ethnicity (non‐Hispanic White, non‐Hispanic Black, Hispanic, non‐Hispanic Others), marital status (married, divorced/widowed/separated, never married), educational attainment (college graduate or more, some college, high school graduate, less than high school), smoking status (never, former, current), year at survey (2000–2004, 2005–2009, 2010–2014, 2015–2018), seen mental health professional past 12 months (yes, no), geographic region (northeast, Midwest, south, west), general health status (excellent/very good, good, fair, poor), and comorbidity score (0, 1–2, ≥ 3). The number of comorbid conditions was determined by compiling responses to several questions regarding the history of conditions such as hypertension, obesity, stroke, coronary heart disease, diabetes, emphysema, kidney disease, and liver diseases (0 representing no and 1 representing yes) [20, 22]. Responses across all conditions were summed to create a comorbidity score and then categorized.

### 2.5 Statistical Analysis

Statistical analysis was performed from February 2024 to May 2024. All analyzes were performed in SAS 9.4 (SAS Institute Inc., Cary, NC) using survey‐specific procedures, which incorporate survey sampling weights to account for the complex sampling design used in the NHIS and to provide representative estimates of the US population. Distribution of the study population characteristics overall and stratified by psychological distress was reported using mean [standard deviation (SD)] and frequency (percentages) where appropriate. Association between covariates and psychological distress was assessed through *χ* ^2^ testing (for categorical variables) and one‐way ANOVA (for continuous variables). Next, multivariable logistic regression was used to estimate the association between psychological distress and ER use adjusting for covariates (age, gender, race/ethnicity, marital status, educational attainment, smoking status, seen mental health professional, general health status, comorbidities, geographic region, year of survey). Finally, multivariable Cox proportional hazards regression was used to model the association between psychological distress and all‐cause mortality adjusting for the same covariates mentioned above. The survival analysis used age at the time of the survey as the timescale, following the recommended approach for household survey‐mortality data linkages [16]. Proportional hazards assumption was tested using the log–log survival and time‐dependent interactions and passed. *p* values were from two‐sided tests, and *p* < 0.05 was determined to be statistically significant.

## 3 Results

**FIGURE 1:**
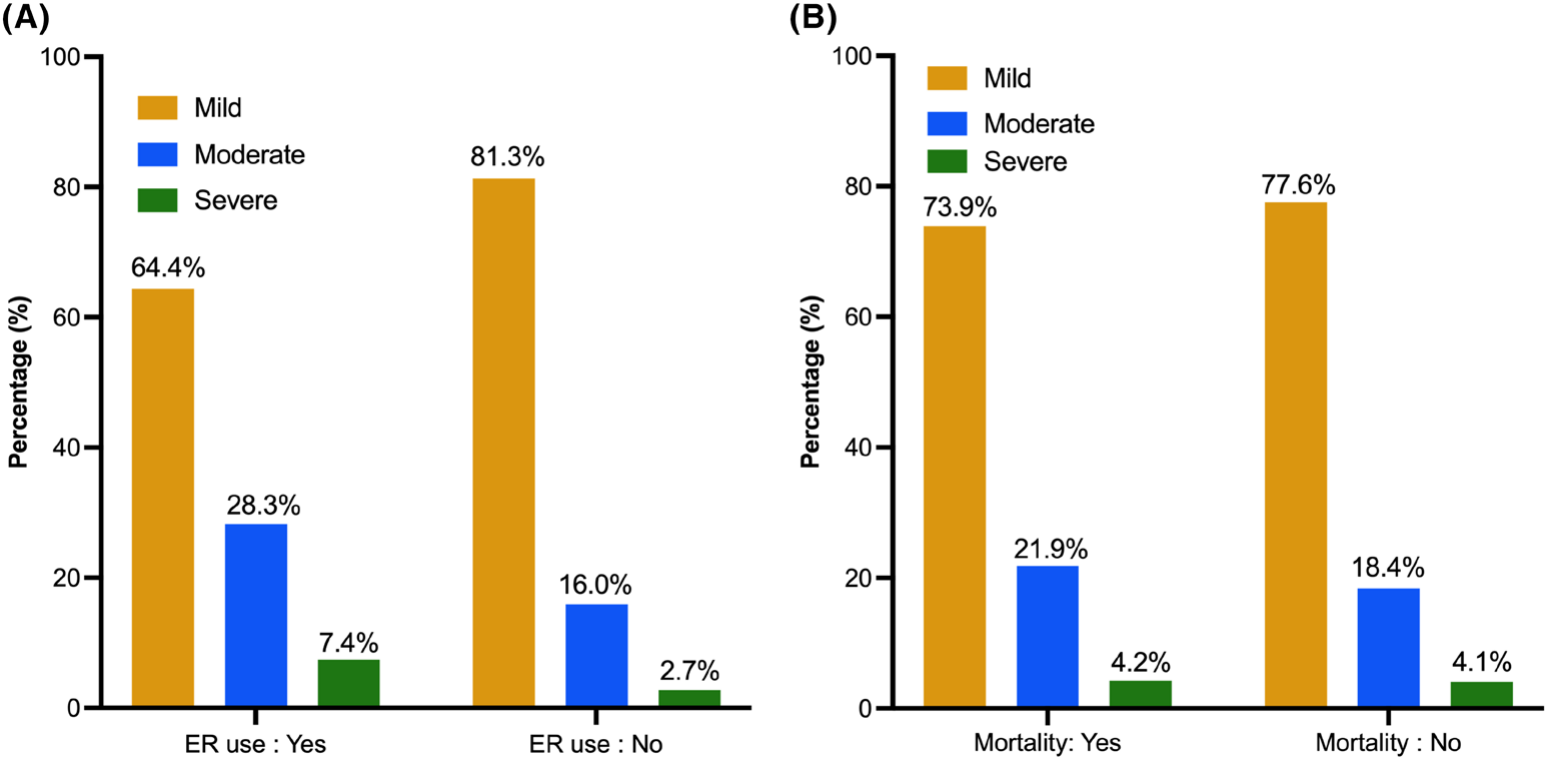
Prevalence of psychological distress by (A) emergency room use and (B) all‐cause mortality (*N* = 3198).

A total of 3198 adults were included in the study; the prevalence of severe and moderate psychological distress was 4.1% and 19.6%, respectively. Approximately 30% of CRC survivors in the study had ER use in the past 12 months, and 41.5% of deaths occurred with a median follow‐up of 84 months. Table 1 presents the demographic characteristics of the study population overall and stratified by psychological distress. Overall, the mean [SD] age at survey was 68 [0.3] years; 51.2% were male, 82.8% were non‐Hispanic Whites, 59.4% were married, 13.2% were current smokers, 21.1% had 3 or more comorbidities, and 6.8% had visited a mental health professional within the past 12 months. In unadjusted analyzes, respondents with severe psychological distress tended to be younger, have lower education (less than a high school diploma), be divorced/widowed/separated, be current smokers, have a higher number of comorbidities, have seen a mental health professional within the past 12 months, and have poor health status. The proportion of CRC survivors reporting moderate or severe psychological distress was higher among those that used the emergency room than those who did not have ER usage (*p* < 0.0001) (Figure 1A). However, the proportion of CRC survivors reporting moderate or severe psychological distress was similar for those that died and those who did not die (*p* = 0.1653) (Figure 1B).

**TABLE 1:** Sample characteristics, overall and stratify psychological distress (*N* = 3198).

Characteristics	Frequency (Weighted %)				*p* ^a^
	Total	Psychological distress			
		No/Mild	Moderate	Severe	
Age at survey, years^b^	68.6 (0.3)	69.6 (0.3)	66.2 (0.8)	62.2 (1.7)	< 0.0001^c^
Time since cancer diagnosis, years^b^	10.3 (0.3)	10.0 (0.2)	11.5 (1.4)	11.8 (1.8)	0.3596^c^
Gender					0.0592
Male	1531 (51.2)	1173 (52.7)	253 (46.0)	66 (47.7)	
Female	1667 (48.8)	1202 (47.3)	350 (54.0)	79 (52.3)	
Race/ethnicity					0.3278
Non‐Hispanic White	2368 (82.8)	1771 (83.2)	444 (82.0)	97 (77.3)	
Others^d^	659 (17.2)	477 (16.8)	135 (18.0)	32 (22.7)	
Educational attainment					< 0.0001
Less than high school	719 (20.1)	478 (17.7)	171 (26.3)	50 (35.2)	
High school graduate	972 (31.9)	721 (31.2)	190 (34.4)	41 (29.8)	
Some college	829 (26.0)	633 (26.7)	148 (24.5)	33 (22.8)	
College graduate or more	647 (22.0)	527 (24.4)	88 (14.8)	18 (12.2)	
Marital status					< 0.0001
Married/living as married	1416 (59.4)	1103 (61.3)	231 (55.5)	47 (41.5)	
Divorced/widowed/separated	1535 (34.1)	1120 (33.4)	306 (34.6)	75 (44.6)	
Never married	247 (6.5)	152 (5.3)	66 (9.9)	23 (13.9)	
Smoking status					< 0.0001
Never	1427 (43.8)	1094 (45.4)	256 (41.1)	54 (36.8)	
Former	1323 (43.0)	1028 (44.8)	227 (39.8)	40 (28.1)	
Current	448 (13.2)	253 (9.8)	120 (19.1)	51 (35.1)	
Year at survey					0.3848
2000–2004	834 (23.5)	624 (24.1)	148 (20.0)	40 (27.6)	
2005–2009	663 (24.7)	498 (24.3)	121 (26.7)	28 (22.3)	
2010–2014	921 (27.2)	675 (27.3)	179 (26.1)	48 (31.1)	
2015–2018	780 (24.6)	578 (24.3)	155 (27.2)	29 (19.0)	
Comorbid conditions^e^					< 0.0001
0	698 (22.7)	558 (24.4)	107 (18.5)	25 (18.6)	
1 to 2	1798 (56.2)	1359 (57.1)	317 (52.3)	76 (50.9)	
3 or more	702 (21.1)	458 (18.5)	179 (29.2)	44 (30.5)	
Seen mental health professional					< 0.0001
Yes	220 (6.8)	97 (4.3)	75 (12.1)	45 (28.6)	
No	2938 (93.2)	2265 (95.7)	525 (87.9)	98 (71.4)	
General health status					< 0.0001
Excellent/very good	987 (32.1)	865 (37.2)	92 (17.6)	14 (8.5)	
Good	1097 (34.8)	890 (37.5)	158 (27.7)	30 (25.2)	
Fair	751 (22.5)	477 (19.5)	210 (33.3)	38 (23.0)	
Poor	358 (10.6)	138 (5.8)	143 (21.4)	63 (43.3)	
Geographic region					0.2866
Northeast	571 (20.6)	426 (20.6)	108 (20.9)	29 (24.6)	
Midwest	800 (25.1)	596 (24.3)	155 (28.8)	25 (16.5)	
South	1176 (36.5)	869 (36.9)	224 (34.9)	58 (38.5)	
West	651 (17.8)	484 (18.2)	116 (15.4)	33 (20.4)	

^a^ *p* calculated using Pearson's chi square test.

^b^ Mean and standard deviation.

^c^ *p* calculated using ANOVA test.

^d^ Others include Non‐Hispanic Black, Non‐Hispanic Asian, Hispanic, Non‐Hispanic other races.

^e^ Comorbid conditions include hypertension, obesity, stroke, coronary heart disease, diabetes, emphysema, kidney disease, and liver disease.

After adjusting for covariates, respondents who experienced moderate [(adjusted odds ratio (aOR) = 1.60, 95% confidence intervals (CI): 1.21–2.10)] or severe (aOR = 1.83, 95% CI: 1.10–3.04) psychological distress were associated with increased odds of ER visits compared to those who experienced no/mild distress (Table 2). In addition to psychological distress, factors such as being divorced/widowed/separated (aOR = 1.43, 95% CI: 1.13–1.80) compared to being married, having three or more comorbid conditions (aOR = 1.61, 95% CI: 1.16–2.23) compared to having no comorbidity, seeing a mental health professional within the past 12 months (aOR = 1.73, 95% CI: 1.17–2.53) compared to not seeing a mental health professional, having fair (aOR = 2.03, 95% CI: 1.49–2.75) or poor general health status (aOR = 5.13, 95% CI: 3.45–7.63) versus excellent health status were associated with increased odds of ER use (Table S1).

**TABLE 2:** Logistic regression model^a^ estimating the association between psychological distress and emergency room use.

Variable	Adjusted odds ratio	95% Confidence interval	*p*
Psychological distress			
No/mild	Reference		
Moderate	1.60	1.21, 2.10	0.0009
Severe	1.83	1.10, 3.03	0.0193

^a^ Model was adjusted for age at survey, time since cancer diagnosis, gender, ethnicity, marital status, education status, smoking status, comorbid conditions, year at survey, mental health professionals, and general health status.

Psychological distress was not significantly associated with all‐cause mortality (Table 3). However, compared to never smokers, respondents who were current smokers (aHR = 2.93, 95% CI: 2.21–3.88) had a higher risk of all‐cause mortality, as did respondents with 3 or more comorbid conditions (1.33, 95% CI: 1.07–1.67) versus no comorbid condition or poor general health status (aHR = 1.69, 95% CI: 1.28–2.23) compared to those with excellent health status (Table S2).

**TABLE 3:** Proportional Cox regression model^a^ estimating the association between psychological distress and all‐cause mortality.

Variable	Adjusted hazard ratio	95% Confidence interval	*p*
Psychological distress			
No/mild	Reference		
Moderate	1.00	0.80, 1.26	0.9878
Severe	1.25	0.80, 1.97	0.3300

^a^ Model was adjusted for time since cancer diagnosis, gender, ethnicity, marital status, education status, smoking status, comorbid conditions, year at survey, mental health professionals, and general health status.

## 4 Discussion

The burden of psychological stressors can profoundly impact CRC survivors, potentially affecting the rate of healthcare utilization. This large nationally representative cross‐sectional study examined NHIS 2000–2018 data to evaluate the prevalence of psychological distress and its association with ER usage and all‐cause mortality in CRC survivors. Among 3198 individuals, 4.1% had severe psychological distress and 19.6% had moderate distress. Moreover, 30% of CRC survivors used ER services and 41.5% of deaths occurred. CRC survivors with severe or moderate psychological distress were more likely to report ER use. However, there was no difference observed across the various psychological distress levels and all‐cause mortality.

There are varying levels of psychological distress reported across the literature among cancer survivors. A previous study focusing on long‐term adult‐onset cancer survivors indicated that 5.6% of the studied population experienced severe psychological distress [23]. Despite using the K6 scale and the 2002–2006 NHIS data, severe psychological distress reported was just slightly higher than what we observed among CRC survivors [23]. Notably, most respondents in their study were survivors of breast, gynecologic, and male genital cancer [23]. This highlights the need for further data to compare CRC to other cancers and to address factors contributing to distress among patients with different cancer diagnoses. Our finding for moderate psychological distress aligns with another study that identified a 17% prevalence of distress using the Brief Symptom Inventory 18 [24]. Meanwhile, Song et al. showed a higher rate of mild to severe psychological distress in CRC survivors undergoing colostomy [25]. They reported distress levels of 96.94% in the first month, 88.55% by the third month, and 29.77% at 6 months post‐procedure. This increased rate of distress can be attributed to the inclusion of mild distress levels, measuring distress within the recent period following the intervention, as well as the specific emphasis on individuals with colostomies. This also shows the acute psychological impact of surgical interventions in patients with CRC, in addition to the challenges associated with caring for a colostomy, which may contribute to the exacerbation of long‐term distress.

We found that ER use was associated with the presence of moderate or severe psychological distress. This is similar to other cancer survivors’ survey evidence demonstrating the role of distress in increased healthcare utilization [10]. CRC cancer survivors having higher moderate or severe psychological distress could be due to different factors. A study by Saunders et al. [26] suggested that failure to effectively manage the psychological symptoms of survivors by primary care services can result in greater healthcare utilization. Abdelhadi showed that cancer survivors experiencing distress were more likely to report low‐quality care, lack of health insurance, limited access to mental health services, and lack of clear communication regarding their treatment plan [10]. These factors might lead to lower adherence to the prescribed medications or recommended treatment, ultimately resulting in poorer clinical outcomes [27]. Additionally, having a negative patient experience coupled with distress may cause delayed seeking of medical attention, leading to the exacerbation of symptoms and increased urgency to visit the ER. It is also crucial to acknowledge different barriers that healthcare team members encounter when assessing psychological distress. These include time constraints, increased workload, limited expertise, and hesitancy to report symptoms by individuals experiencing distress. Herein, the role of tailored interventions can be of high value to bridge the gap by enhancing patient access to psych‐oncology care and providing training for team members on recognizing red flags and key symptoms that necessitate assessment and referrals to supportive services [28]. For instance, a study investigating the differences in psychological distress among postoperative chemotherapy patients with CRC found that the implementation of mindfulness intervention compared to mindfulness intervention combined with homogeneous medical concepts played a role in alleviating and improving patients emotions and quality of life [29].

Socioeconomic factors can impact individuals' levels of psychological distress. Among CRC survivors in this study, severe distress was commonly observed among participants who were younger and those who were divorced, widowed, or separated. Similar age‐related findings were reported by another study [10]; it was also previously indicated that individuals with lower income [10] or unemployment [24] had a higher prevalence of distress. Furthermore, Ramp et al. [30], showed that psychological distress was significantly associated with the need for dietary support among CRC survivors. Therefore, it is important to adopt a holistic view of cancer survivors beyond their physical health, taking into consideration cues from their mental and social screening. It is also essential to address barriers to healthcare access among underserved minorities and to factor in the presence of a social support system in their psychological assessment.

Previous studies highlighted the feasibility and cost‐effectiveness of distress screening within the target population [31, 32, 33]. Integrating distress screening across the service line requires a multi‐level approach to ensure equitable access to care. This could involve operational changes within the system, ensuring the availability of resources for healthcare professionals to act upon clinically significant patient symptoms, and encouraging patients' adoption and access to the offered distress assessment and treatment tools.

This study has limitations. First, due to its cross‐sectional study design, we were unable to draw causal inferences. Therefore, it is suggested that a longitudinal evaluation of survivors would be advantageous for a deeper understanding of individualized factors influencing psychological distress. Second, despite the precision, sensitivity, and specificity of the K6 scale, it has not been validated specifically for CRC survivors. This may result in overlooking some psychological symptoms and possibly undermining the actual rate of distress. Third, the lack of information regarding the type of psychological disorder, cancer stage, and presence of a stoma within the used database fails to capture important data that have been shown to impact levels of distress. Future research should account for these variables as they would provide a comprehensive understanding of the disease and help inform tailored intervention. Finally, the mortality status depended on NHIS reported 20‐year follow‐up data, which may have led to misclassifying respondents survival, thereby affecting the analysis.

## 5 Conclusion

One‐in‐four CRC survivors in our study reported severe or moderate psychological distress. CRC survivors with severe or moderate psychological distress were more likely to report ER use. This finding emphasizes the significance of timely identification and intervention for psychological distress to improve quality of life and long‐term clinical outcomes of patients diagnosed with CRC. Integrating mental health support into routine cancer care may reduce distress levels, potentially leading to fewer ER usages among CRC survivors. A multidimensional approach should be adopted to implement distress screening and address existing system‐, physician‐, and patient‐related barriers. Finally, individuals' social factors could be taken into consideration to enhance patient‐centered care and facilitate personalized interventions.

## Author Contributions

**Pranali G. Patel:** conceptualization, investigation, writing – original draft, methodology, validation, formal analysis, data curation. **Chaitali Dagli:** data curation, methodology, validation, conceptualization, investigation, writing – original draft. **Nada Al‐Antary:** methodology, data curation, investigation, validation, writing – original draft. **Mrudula Nair:** methodology, validation, data curation, writing – original draft. **Oluwole A. Babatunde:** methodology, investigation, validation, writing – review and editing. **Nosayaba Osazuwa‐Peters:** writing – review and editing, methodology, validation, investigation. **Poolakkad S. Satheeshkumar:** methodology, validation, investigation, writing – review and editing. **Eric Adjei Boakye:** conceptualization, investigation, writing – original draft, methodology, validation, writing – review and editing, formal analysis, data curation, supervision.

## Disclosure

Prior meeting presentation: The abstract of the manuscript was presented as a poster at the 17th AACR Conference on the Science of Cancer Health Disparities in Racial/Ethnic Minorities and the Medically Underserved; 2024 September 21–24; Los Angeles, CA, USA.

## Ethics Statement

This study used the publicly available dataset—National Health Interview Survey (NHIS). This NHIS was approved by the National Center for Health Statistics Ethics Review Board and conducted in accordance with the Declaration of Helsinki.

## Consent

Survey respondents provided verbal consent.

## Conflicts of Interest

Nosayaba Osazuwa‐Peters declare(s) personal fees from Navigating Cancer and Merck. The remaining authors declare no conflicts of interest.

## Supporting information


**Data S1:** cam471107‐sup‐0001‐Supinfo1.docx.

## Data Availability

The data is publicly available and could be assessed at the NHIS website: https://www.cdc.gov/nchs/nhis/documentation/index.html. Additional requests for analytical datasets used in this study may be sent to the corresponding author.
