# Supplementary material for: The Association Between Psychological Distress, Emergency Room Visits, and All‐Cause Mortality Among Colorectal Cancer Survivors
Source: Cancer Med. 2025 Jul 29;14(15):e71107. doi: 10.1002/cam4.71107 (PMC12307967; doi:10.1002/cam4.71107)
Supplement: Supplementary file 1 — Data S1: cam471107‐sup‐0001‐Supinfo1.docx. [file CAM4-14-e71107-s001.docx]

**Supplemental Table 1: Logistic regression model examining factors associated with emergency room use**

|  | **aOR** | **95% CI** | **p-value** |
| --- | --- | --- | --- |
| **Age at survey, years** | 1.00 | 0.99, 1.01 | 0.9114 |
| **Time since cancer diagnosis, years** | 1.00 | 0.99, 1.01 | 0.4979 |
| **Gender** |  |  |  |
| Female | Reference | | |
| Male | 1.10 | 0.87, 1.39 | 0.4258 |
| **Race/ethnicity** |  |  |  |
| Non-Hispanic White | Reference | | |
| Others^a^ | 0.90 | 0.69, 1.17 | 0.4231 |
| **Educational attainment** |  |  |  |
| College graduate or more | Reference | | |
| Less than high school | 0.87 | 0.63, 1.22 | 0.4172 |
| High school graduate | 0.90 | 0.66, 1.24 | 0.5287 |
| Some college | 1.22 | 0.89, 1.66 | 0.2134 |
| **Marital status** |  |  |  |
| Married/living as married | Reference | | |
| Divorced/Widowed/Separated | 1.43 | 1.13, 1.80 | 0.0026 |
| Never married | 0.93 | 0.62, 1.39 | 0.7112 |
| **Smoking status** |  |  |  |
| Never | Reference | | |
| Former | 1.03 | 0.82, 1.30 | 0.7293 |
| Current | 0.87 | 0.60, 1.27 | 0.4824 |
| **Year at survey** |  |  |  |
| 2000-2004 | Reference | | |
| 2005-2009 | 1.02 | 0.76, 1.36 | 0.9225 |
| 2010-2014 | 0.95 | 0.72, 1.26 | 0.7245 |
| 2015-2018 | 0.95 | 0.71, 1.28 | 0.7373 |
| **Comorbid conditions^b^** |  |  |  |
| 0 | Reference | | |
| 1 to 2 | 1.08 | 0.81, 1.43 | 0.6025 |
| 3 or more | 1.61 | 1.16, 2.23 | 0.0040 |
| **Seen mental health professional** |  |  |  |
| No | Reference | | |
| Yes | 1.73 | 1.17, 2.53 | 0.0055 |
| **General health status** |  |  |  |
| Excellent/very good | Reference | | |
| Good | 1.41 | 1.07, 1.85 | 0.0140 |
| Fair | 2.03 | 1.49, 2.75 | <0.0001 |
| Poor | 5.13 | 3.45, 7.63 | <0.0001 |
| **Geographic region** |  |  |  |
| South | Reference | | |
| Northeast | 1.22 | 0.90, 1.65 | 0.2057 |
| Midwest | 1.09 | 0.84, 1.42 | 0.5182 |
| West | 1.11 | 0.83, 1.48 | 0.4872 |
| Model was adjusted for psychological distress  ^a^Others – Non-Hispanic Black, Non-Hispanic Asian, Hispanic, Non-Hispanic other races  ^b^Comorbid conditions - hypertension, obesity, stroke, coronary heart disease, diabetes, emphysema, kidney disease, and liver diseases | | | |

**Supplemental Table 2: Cox Proportional regression model examining factors associated with all-cause mortality**

|  | **aHR** | **95% CI** | **p-value** |
| --- | --- | --- | --- |
| **Time since cancer diagnosis, years** | 0.99 | 0.98, 1.00 | 0.1534 |
| **Gender** |  |  |  |
| Female | Reference | | |
| Male | 1.09 | 0.90, 1.31 | 0.3793 |
| **Race/ethnicity** |  |  |  |
| Non-Hispanic White | Reference | | |
| Others^a^ | 1.13 | 0.95, 1.34 | 0.1651 |
| **Educational attainment** |  |  |  |
| College graduate or more | Reference | | |
| Less than high school | 0.91 | 0.73, 1.13 | 0.3724 |
| High school graduate | 1.17 | 0.94, 1.46 | 0.1686 |
| Some college | 1.13 | 0.90, 1.40 | 0.2898 |
| **Marital status** |  |  |  |
| Married/Living as married | Reference | | |
| Divorced/Widowed/Separated | 0.66 | 0.55, 0.78 | <0.0001 |
| Never married | 1.17 | 0.81, 1.69 | 0.3945 |
| **Smoking status** |  |  |  |
| Never | Reference | | |
| Former | 1.23 | 1.03, 1.45 | 0.0189 |
| Current | 2.93 | 2.21, 3.88 | <0.0001 |
| **Year at survey** |  |  |  |
| 2000-2004 | Reference | | |
| 2005-2009 | 0.71 | 0.59, 0.85 | 0.0002 |
| 2010-2014 | 0.45 | 0.38, 0.54 | <0.0001 |
| 2015-2018 | 0.16 | 0.12, 0.21 | <0.0001 |
| **Comorbid conditions^b^** |  |  |  |
| 0 | Reference | | |
| 1 to 2 | 1.00 | 0.83, 1.21 | 0.9770 |
| 3 or more | 1.33 | 1.07, 1.67 | 0.0117 |
| **Seen mental health professional** |  |  |  |
| No | Reference | | |
| Yes | 1.55 | 1.08, 2.16 | 0.0121 |
| **General health status** |  |  |  |
| Excellent/very good | Reference | | |
| Good | 1.11 | 0.93, 1.32 | 0.2473 |
| Fair | 1.22 | 1.01, 1.47 | 0.0443 |
| Poor | 1.69 | 1.28, 2.23 | 0.0002 |
| **Geographic region** |  |  |  |
| South | Reference | | |
| Northeast | 0.75 | 0.61, 0.93 | 0.0091 |
| Midwest | 0.87 | 0.71, 1.06 | 0.1719 |
| West | 0.92 | 0.77, 1.09 | 0.3405 |
| Model was adjusted for psychological distress  ^a^Others – Non-Hispanic Black, Non-Hispanic Asian, Hispanic, Non-Hispanic other races  ^b^Comorbid conditions - hypertension, obesity, stroke, coronary heart disease, diabetes, emphysema, kidney disease, and liver diseases | | | |
